# Preoperative Planning for Reverse Shoulder Arthroplasty: Does the Clinical Range of Motion Match the Planned 3D Humeral Displacement?

**DOI:** 10.3390/jpm13050771

**Published:** 2023-04-29

**Authors:** Diane Ji Yun Yoon, Guillaume-Anthony Odri, Luc Favard, Ramy Samargandi, Julien Berhouet

**Affiliations:** 1 Service de Chirurgie Orthopédique et Traumatologique, CHRU Trousseau, Faculté de Médecine de Tours, Université de Tours, 1C Avenue de la République, 37170 Chambray-les-Tours, France; 2 Inserm U1132 BIOSCAR, Université Paris Cité, 75010 Paris, France; 3 Service de Chirurgie Orthopédique et Traumatologique, Centre Hospitalier Universitaire Lariboisière, 75010 Paris, France; 4 Department of Orthopedic Surgery, Faculty of Medicine, University of Jeddah, Jeddah 23218, Saudi Arabia; 5 Equipe Reconnaissance de Forme et Analyse de l’Image, Laboratoire d’Informatique Fondamentale et Appliquée de Tours EA6300, Ecole d’Ingénieurs Polytechnique Universitaire de Tours, Université de Tours, 64 Avenue Portalis, 37200 Tours, France

**Keywords:** reverse shoulder arthroplasty, range of motion, preoperative planning, arm change position, motion analysis

## Abstract

Introduction: The functional outcome after reverse shoulder arthroplasty (RSA) is closely linked to how much the humerus shifts because of the implants. While two-dimensional (2D) angle measurements have been used to capture this shift, it can be measured in three dimensions (3D) as the arm change position (ACP). In a previous study, the ACP was measured using 3D preoperative planning software with the passive virtual shoulder range of motion obtained after RSA. The main objective of this study was to evaluate the relationship between the ACP and the actual active shoulder range of motion measured after RSA. The hypothesis was that the ACP and the active clinical range of motion are related such that the ACP is a reliable parameter to guide the preoperative planning of an RSA. The secondary objective was to assess the relationship between 2D and 3D humeral displacement measurements. Materials and methods: This prospective observational study enrolled 12 patients who underwent RSA and had a minimum follow-up of 2 years. The active range of motion in shoulder flexion, abduction, and internal and external rotation was measured. At the same time, ACP measurements were taken from a reconstructed postoperative CT scan, in addition to the radiographic measurements of humeral lateralization and distalization angles on AP views in neutral rotation. Results: The mean humeral distalization induced by RSA was 33.3 mm (±3.8 mm). A non-statistically significant increase in shoulder flexion was observed for humeral distalization beyond 38 mm (R^2^ = 0.29, *p* = 0.07). This “threshold” effect of humeral distalization was also observed for the gains in abduction, as well as internal and external rotations, which seemed better with less than 38 mm or even 35 mm distalization. No statistical correlation was found between the 3D ACP measurements and 2D angle measurements. Conclusion: Excessive humeral distalization seems to be detrimental to joint mobility, especially shoulder flexion. Humeral lateralization and humeral anteriorization measured using the ACP seem to promote better shoulder range of motion, with no threshold effect. These findings could be evidence of tension in the soft tissues around the shoulder joint, which should be taken into consideration during preoperative planning.

## 1. Introduction

Reverse total shoulder arthroplasty (RSA) has shown excellent functional outcomes and therefore has become widely accepted as the treatment of choice for cuff tear arthropathy [1]. In recent years, the indications of RSA have been expanded for the treatment of various conditions, including pseudoparalysis due to massive irreparable rotator cuff tears, acute three-part or four-part proximal humerus fractures, revision arthroplasty, chronic irreducible shoulder dislocation, tumor resection, and inflammatory joint conditions such as rheumatoid arthritis [2,3,4,5,6,7,8,9]. In the last decade, numerous lines of evidence demonstrated that RSA is a safe and effective treatment in patients younger than 65 years, which offers a substantial functional improvement in pain level, range of motion, and strength [10,11,12,13]. Previous studies reported a 10-year survival of 91.0% in case of primary RSA and 80.9% in case of revision RSA for failed arthroplasty [12]. The Australian National Joint Registry reported similar results, with a 10-year survival for the diagnosis of rotator cuff arthropathy of 94.1% [14]. The incidence of primary RSA in the United States has increased from 22,835 procedures in 2011 to 62,705 procedures in 2017. The greatest increase in incidence was found in male patients and patients aged between 50 and 64 years, whereas the incidence of hemiarthroplasty has markedly decreased in recent years [15]. In France, primary shoulder arthroplasty procedures increased by 47% between 2012 and 2018 and are estimated to grow from 31% to 322% between 2018 and 2050 [16].

An RSA design was first proposed by Paul Grammont in 1985 to lower and shift the humerus medially [17,18]. Since the deltoid’s lever arm increases, the shoulder’s mobility in flexion and abduction (ABD) improves [3,19]; however, rotation with the elbow at the side is often deficient [20,21]. Scapular notching is another limitation of this humeral medialization [20,22]. Scapular notching was described by Sirveaux et al. and was theorized to be caused by the mechanical impingement of the medial side of the humeral polyethylene component against the inferior scapular neck [23]. Scapular notching can be avoided by the lateralization of the center of rotation either through a lateralized RSA design or bony increased-offset RSA [20,24,25].

At the same time, excessive arm lengthening can lead to neurological impairment [26,27]. Thus, it is hard to grasp how each component of humeral displacement impacts the functional outcomes after RSA.

Boutsiadis et al. [28] recently described two radiographic angle measurements: “lateralization shoulder angle” (LSA) and “distalization shoulder angle” (DSA). These evaluate humeral lateralization and distalization, respectively, induced by the RSA. The main limitation of these angles is that they are measured with anteroposterior X-ray views, in a two-dimensional (2D) frame of reference. Additionally, the quality of the images affects the measurement accuracy. 

Berhouet et al. [29] recently described another parameter, called the “arm change position” (ACP), to evaluate humeral displacement after RSA but this time in three dimensions (3D). The ACP is calculated using a 3D preoperative planning software program for the placement of shoulder replacement implants. In that study, the virtual range of motion (RoM) analysis and the ACP measurement of humeral displacement after the RSA showed that lowering and shifting the humerus laterally improved shoulder mobility in flexion–extension, external rotation with the elbow at the side (ER1), and adduction (ADD). Better internal rotation with the elbow at the side (IR1) was also found when the humerus was shifted anteriorly [29]. Nevertheless, this virtual analysis was somewhat unrealistic because it did not consider how the scapulothoracic joint contributes to the shoulder joint’s movements. The Scapulothoracic participation plays a more important role in RSA biomechanics than in normal shoulder biomechanics [30,31]. 

The main objective of this study was to determine the relationship between 3D humeral displacements based on the ACP after RSA and the true active RoM measured in a clinical setting. If these are found to be related, we hypothesize that the ACP is a reliable parameter to guide the choice and positioning of RSA implants during preoperative planning. The study’s secondary objective was to determine the relationship between the humeral displacements measured in 3D using the ACP and those in 2D using the LSA and DSA.

## 2. Materials and Methods

### 2.1. Materials

#### 2.1.1. Patients

**Figure 1:**
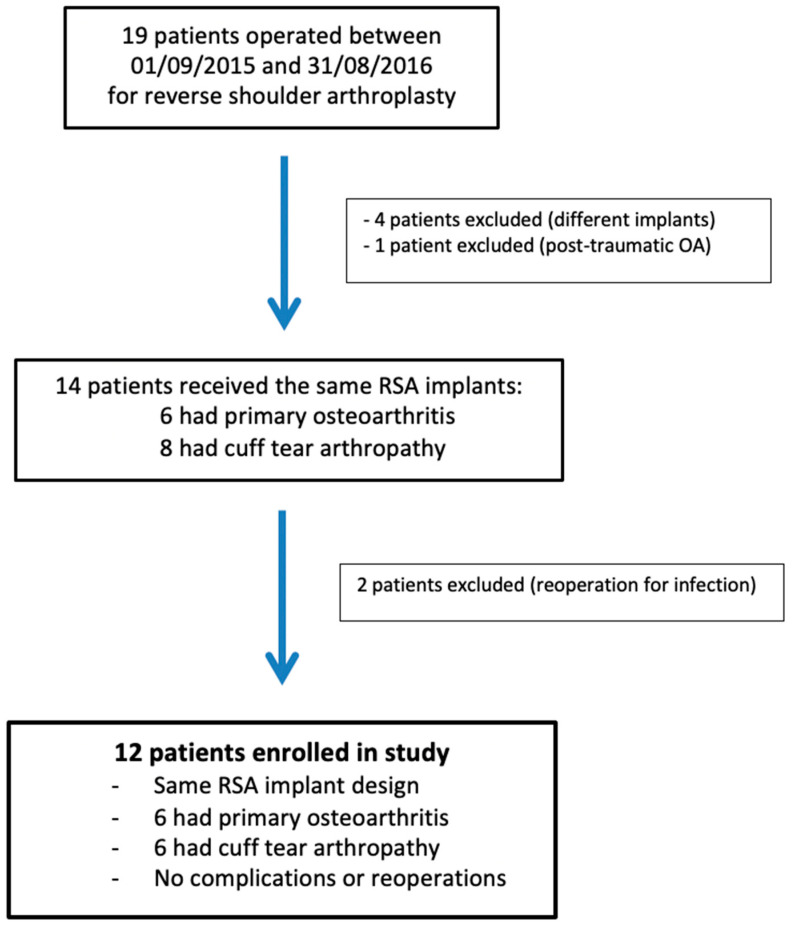
Flowchart of patients included in the study. RSA: reverse shoulder arthroplasty; OA: osteoarthritis.

The research protocol approval was obtained from our institutional review board (IRB: 13B-T-SHOULDER-RM). This prospective observational study enrolled 12 patients (9 women and 3 men), with a mean age of 73 ± 4.3 years (range, 63 to 81 years), who had undergone RSA surgery between 9 January 2015 and 31 August 2016 (Figure 1). RSA indications were primary osteoarthritis and cuff tear arthropathy (CTA), with six patients in each group. Their average body mass index was 28.5 ± 3 kg/m^2^ (19.5–34.4). RSA was performed on the dominant side in nine cases. The shoulder undergoing RSA had never been operated on. The preoperative demographic, clinical, and imaging data of the study population are presented in Table 1.

#### 2.1.2. RSA Implants

A reversed shoulder prosthesis (Wright Medical France, Monbonnot Saint Martin, France) was used on all shoulders. The humeral stem was an AEQUALIS ASCEND™ FLEX system (Tornier, Bloomington, MN, USA), which has a 132.5° fixed inclination Onlay stem mounted with a 1.5 mm to 3.5 mm lateralized humeral plateau with variable indexing and a standard polyethylene insert having a fixed tilt angle of 12.5°. The final humeral configuration had a 145° inclination. 

The glenosphere implant was Reversed AEQUALIS™ (Tornier, Bloomington, MN, USA). It included a 25 mm (*n* = 7) or 29 mm (*n* = 5) diameter baseplate with a short or long peg and a 36 mm (*n* = 9) or 42 mm (*n* = 3) centered sphere. A 7–10 mm thick lateralizing glenoid bone graft (BIO-RSA) was added in nine patients.

#### 2.1.3. Software

**Figure 2:**
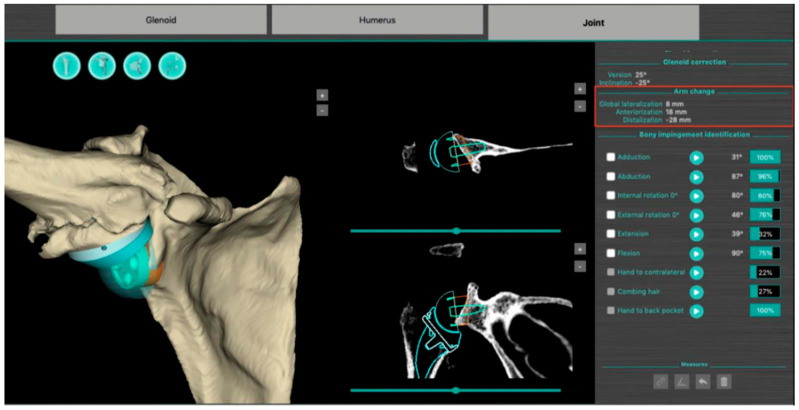
Screenshot of a preoperative plan for RSA from the BluePrint^®^ 3D Planning software (Stryker, Monbonnot Saint Martin, France). In addition to a virtual analysis of the passive joint range of motion, information on humeral positioning (arm change position) is included (red frame).

**Figure 3:**
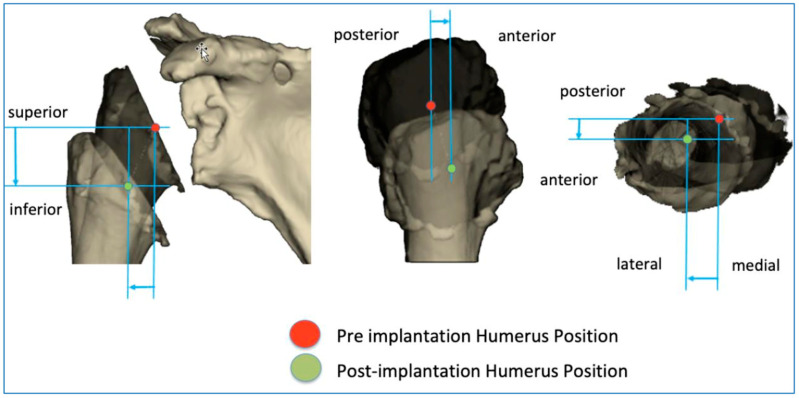
Illustration of how the arm change position (ACP) is measured in three dimensions.

The BluePrint^®^ 3D Planning software (Tornier SAS France, Monbonnot Saint Martin, France) was used to select the type of implants to use in a given patient and to position them in a virtual shoulder joint. Once the implant configuration is finalized, the software simulates the passive RoM and measures humeral displacement in the frontal, sagittal, and axial planes. This is the ACP parameter (expressed in millimeters), which comprises 3 values that correspond to the superior–inferior, mediolateral, and anteroposterior humeral displacements after the virtual implantation of the RSA (Figure 2). These three values are positive when the displacement is superior, lateral, and anterior. They are negative when the displacement is inferior, medial, and posterior (Figure 3).

Postoperatively, another software package (PTC Creo^®^ version 6.0, Parametric Technology Corporation, Needham, MA, USA) was used to capture a postoperative CT scan of the patient’s shoulder with the implants in place. These images were uploaded into the Blueprint software to measure the ACP postoperatively, as this corresponds to the actual implant positioning.

### 2.2. Methods

The preoperative imaging assessment included standard AP X-rays in three different rotations plus an axillary view and a CT scan of the shoulder for 3D planning purposes. All the patients were operated on using the deltopectoral approach for the implantation of the prosthesis, in a beach chair position and under general anesthesia combined with an interscalene block. The procedure was performed in a standard manner with no particular technical point during the procedure. The retroversion of the humeral implant was adjusted relative to the forearm axis and ranged from 10° to 30°, depending on the patient. The subscapularis was repaired when it was still present. Biceps tenodesis to the pectoralis major was carried out at the end of the procedure. The patients were discharged 48 h after surgery. Postoperative care included shoulder immobilization with an abduction pillow for 3 to 6 weeks. Physiotherapy was started immediately or at 3 weeks if BIO-RSA had been performed.

The 12 patients were reviewed at a minimum follow-up of 2 years postoperatively. Their active shoulder joint mobility was measured in degrees for flexion (F), abduction (ABD), external rotation (ER1/ER2) with the elbow at the side/with the arm abducted at 90°, and internal rotation (IR1) with the elbow at the side, as it had been during the preoperative consultation. The active internal rotation range of motion measurement was defined as the highest midline vertebral segment of the back that can be reached. This measurement was converted into a 10-point scale according to the Constant–Murley Shoulder Outcome Score guidelines [32]. 

**Figure 4:**
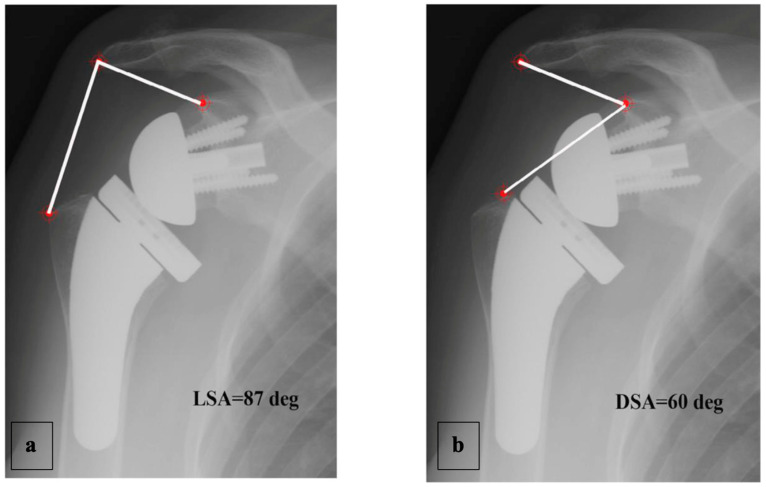
Example of LSA (**a**) and DSA (**b**) measured on an AP radiograph in neutral shoulder rotation, as described by Boutsiadis et al. [12].

A complete radiographic assessment identical to that carried out preoperatively (AP view in three rotations and axillary view) was performed; fluoroscopy was used beforehand to ensure the images were reproducible between the patients. The LSA and DSA in degrees (°) were measured on AP views in neutral rotation, as described by Boutsiadis et al. [12] (Figure 4).

**Figure 5:**
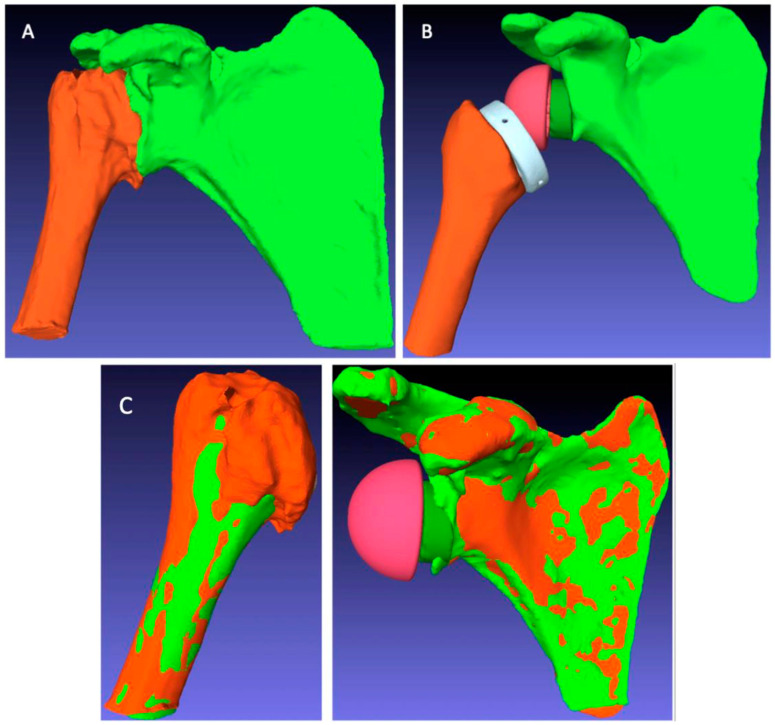
Image processing steps for postoperative arm change position (ACP) measurement: (**A**) automatic segmentation to extract the humeral and scapular bones from the postoperative CT scan; (**B**) manual segmentation to extract the bones and implants from the postoperative CT scan; (**C**) registration overlay of the preoperative (green) and postoperative (orange) structures to measure the ACP corresponding to the postoperative situation.

Finally, at the same minimum follow-up of 2 years, a CT scan of the operated shoulder was carried out to measure the ACP after RSA using the BluePrint^®^ planning software. However, the presence of the implants meant that these CT scans were not directly supported by the BluePrint^®^ software. Several image preparation and processing steps had to be completed before the postoperative ACP could be measured in each patient (Figure 5).

These processing steps were as follows:

1. The extraction of the 3D geometry of the humerus and scapula from the preoperative CT scan;
2. The manual registration of the preoperative 3D geometry of the humerus and scapula, with RSA implants from the postoperative CT scan, using the PTC Creo^®^ software;
3. The creation of planning files integrating the readjusted bone and implant geometries in the BluePrint^®^ software to measure the postoperative ACP corresponding to the RSA implant configuration specific to each patient.

The data were collated in an Excel^®^ spreadsheet and analyzed using the JMP^®^ 11.0.0 software (SAS Institute Inc.©, Cary, NC, USA). A Shapiro–Wilk test was used to evaluate the normal distribution of the continuous quantitative variables. A Student’s *t*-test was performed to compare the means between indication subgroups; linear regression was carried out for the correlations. The significance level was set at *p* < 0.05.

## 3. Results

### 3.1. Descriptive Data

RSA improved the active shoulder RoM in all patients. These improvements were similar for the two groups (Table 2). Internal rotation was unchanged in the CTA group, whereas it was improved in the primary osteoarthritis group relative to the preoperative measurement. The other RoM (ΔF, ΔABD, ΔER1, and ΔER2) had also further increased compared with the preoperative RoM for the primary osteoarthritis group (Table 3).

The mean humeral distalization induced by RSA, assessed using the ACP, was 33.3 mm (±3.8 mm). The mean lateralization was 4.3 mm (±3.7 mm). The mean anterior displacement of the humerus (“anteriorization”) was 6 mm (±6 mm). There was no significant difference between the indications for these three parameters (Table 2). The mean LSA and DSA values were 80.9° (±11.1°) and 48.5° (±9.7°), with no difference between the two indications (Table 2).

### 3.2. Analytical Data

**Figure 6:**
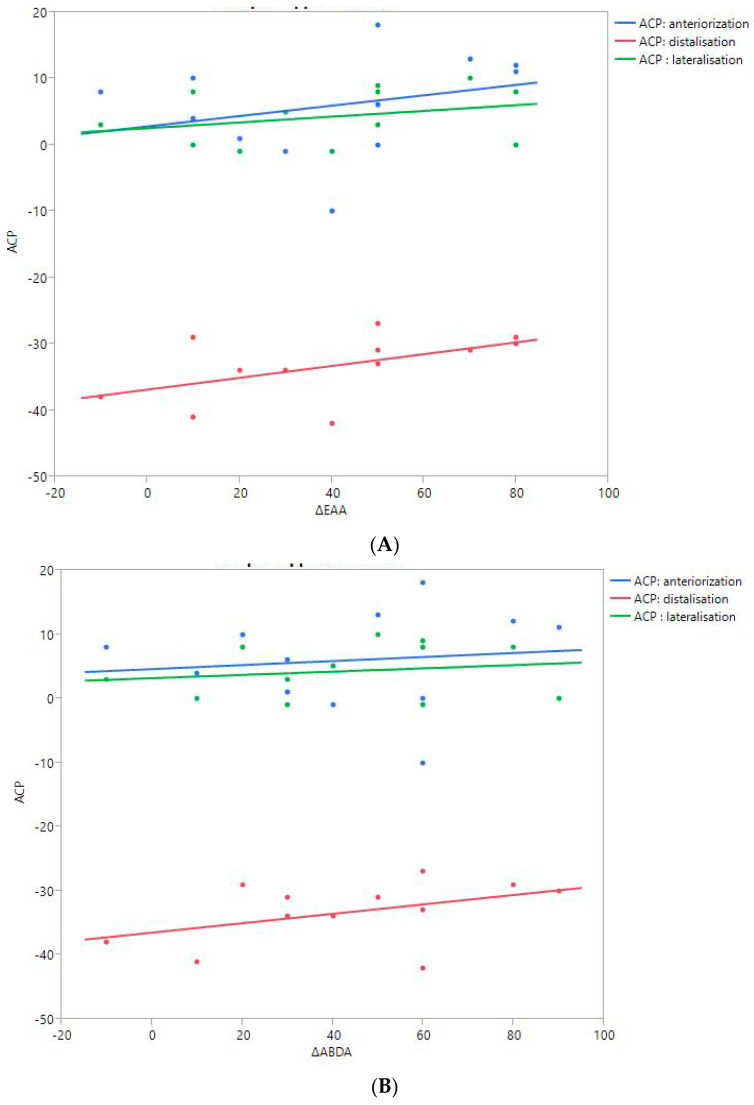

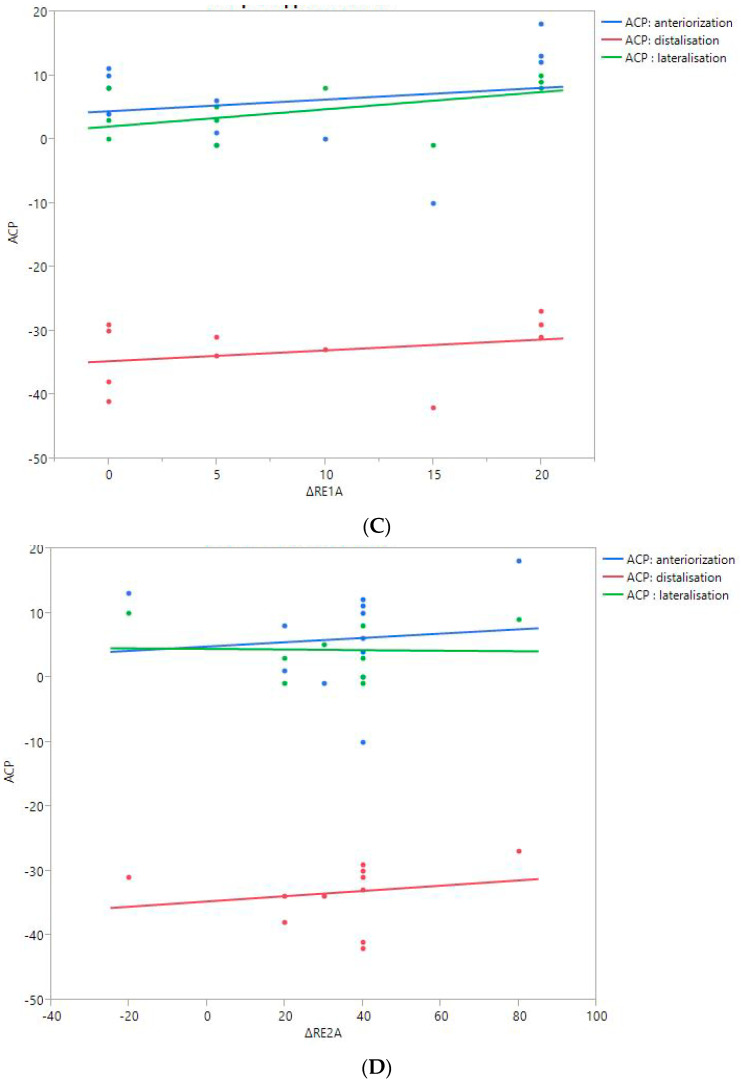

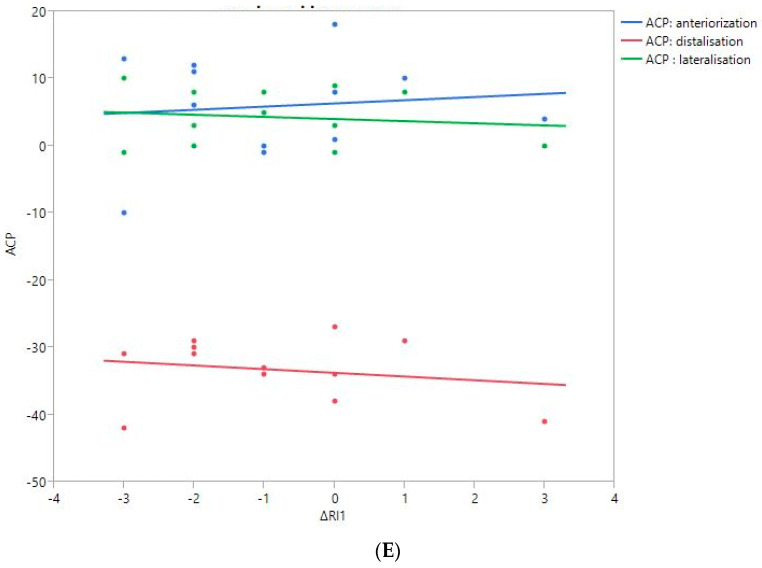
Shoulder motion and 3D humeral displacement according to the arm change position (ACP). (**A**) Change in active shoulder flexion amplitude (Δ F) relative to ACP. (**B**) Change in active abduction amplitude (Δ ABD) relative to ACP. (**C**) Change in active external rotation elbow at side (Δ RE1A) relative to ACP. (**D**) Change in active external rotation with 90° shoulder abduction (Δ RE2A) relative to ACP. (**E**) Change in active internal rotation elbow at side (Δ RI1) relative to ACP.

RoM and Measurement of 3D Humeral Displacement Using the ACP (Figure 6).

There were no statistically significant findings; however, there were two interesting trends observed:

- The first observation was the gains in flexion. Flexion tended to decrease with humeral distalization (or lowering). There was a threshold around 38 mm, which was associated with the worse RoM values; shoulder flexion was better when the resulting humerus position was less distal (R^2^ = 0.29, *p* = 0.07). This “threshold” effect of humeral distalization was also observed for other shoulder motions, but the correlation was not as strong. The gains in ER1, ABD, ER2, and IR1 seemed better when the humerus was lowered less than 35 or 38 mm, depending on the patient.
- The second observation was the gains in ER1, which tended to improve with humeral lateralization (R^2^ = 0.29, *p* = 0.07).

Among our other results, a positive linear relationship was found between the improvement in ABD, F, IR1, and ER2 and the anterior humeral displacement. Humeral lateralization was associated with a slightly worse range of motion in ER2 and IR1.

**Figure 7:**
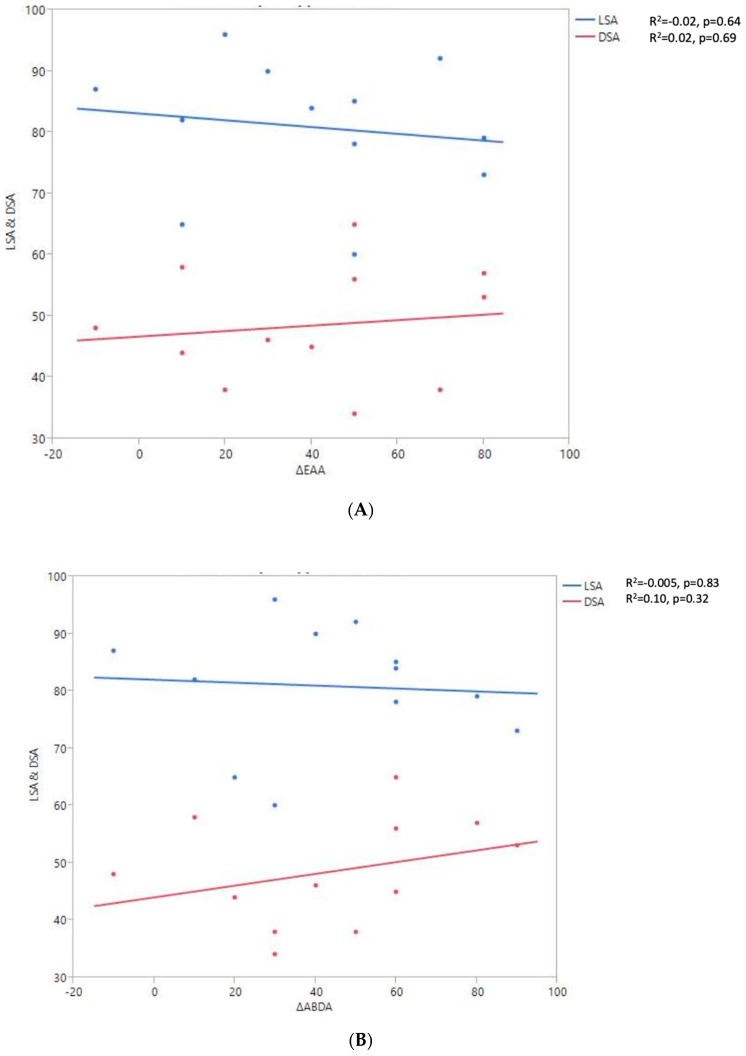

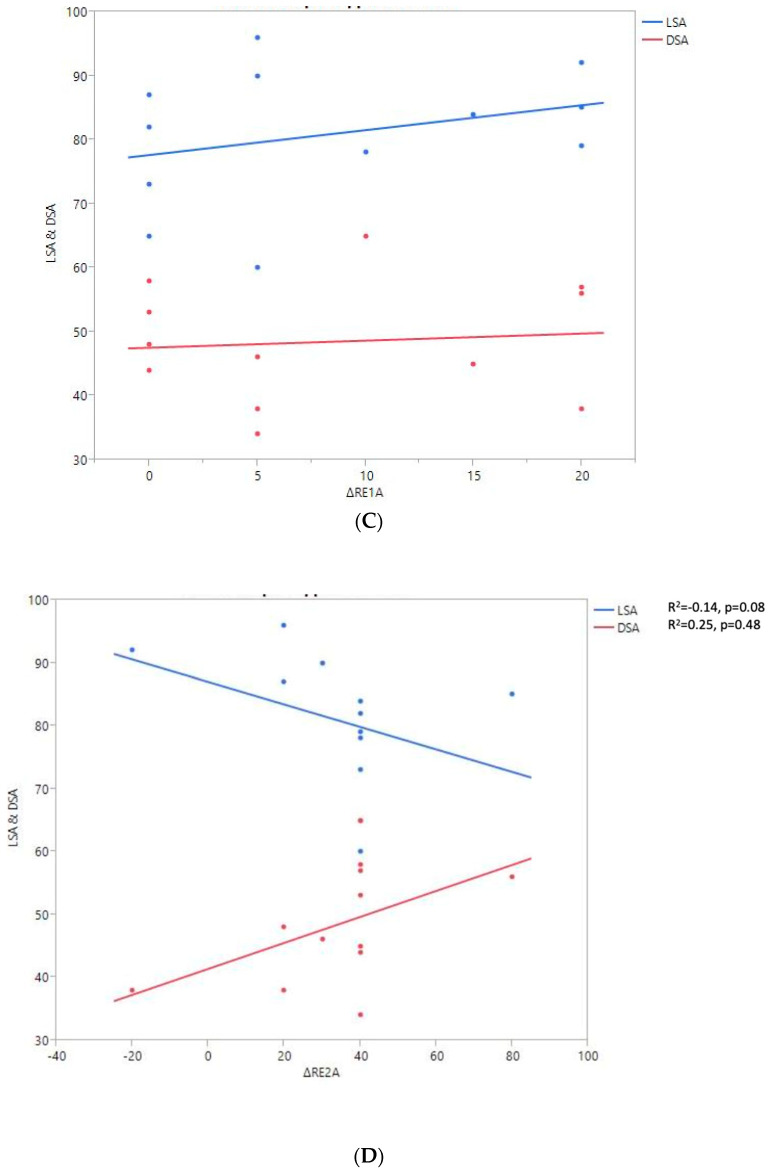

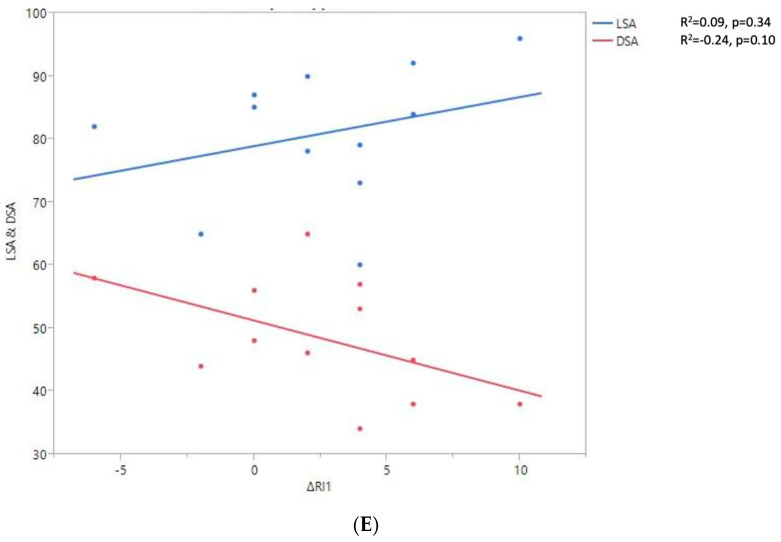
Shoulder motion and 2D humeral displacement using DSA and LSA. (**A**) Change in active shoulder flexion amplitude (Δ F) relative to DSA and LSA. (**B**) Change in active abduction (Δ ABD) relative to DSA and LSA. (**C**) Change in active external rotation elbow at side (Δ RE1A) relative to DSA and LSA. (**D**) Change in active external rotation with arm in abduction (Δ RE2A) relative to DSA and LSA. (**E**) Change in active internal rotation elbow at side (Δ RI1) relative to DSA and LSA.

RoM and Measurement of 2D Humeral Displacement Using the DSA and LSA (Figure 7).

A positive linear regression was observed between F, ABD, ER1, ER2, and humeral distalization assessed using the DSA. On the other hand, the IR1 decreased when the DSA increased. The greater humeral lateralization assessed using the LSA was associated with better ER1 and IR1 but worse F and ER2. However, none of these findings were statistically significant.

**Figure 8:**
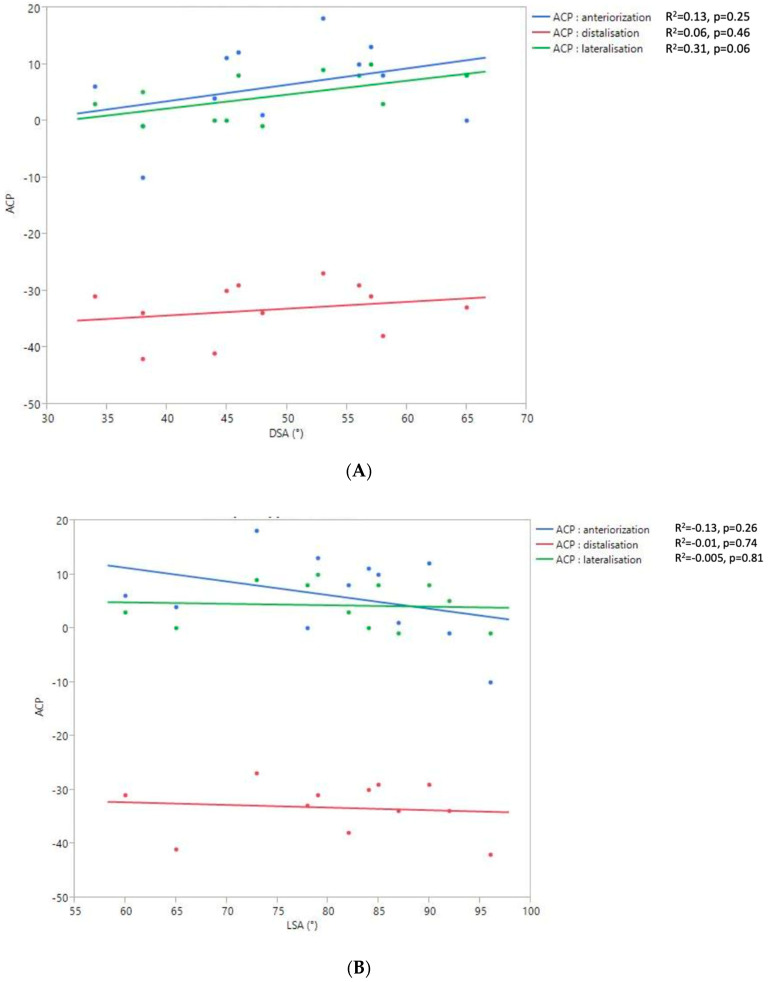
Relationship between the 2D DSA (**A**) and 2D LSA (**B**) measurements relative to the 3D ACP measurement.

The 2D and 3D Humeral Displacement Measurements (Figure 8).

No correlation was found between the measurements of 3D humeral displacement using the ACP and 2D displacement using the LSA and DSA.

## 4. Discussion

Restoring active shoulder flexion is one of the main functional objectives of RSA. In this clinical study, the main finding was that excessive humeral distalization (or lowering) after RSA implantation somewhat reduced flexion amplitude. This observation was made based on the 3D and 2D analyses of humeral displacement. The mean distalization ACP was 33.3 ± 3.8 mm, and the flexion amplitude was better for the smallest ACP values. The mean DSA was 48.5 ± 9.7°, and the flexion amplitude was also better for the smallest DSA values. Werner et al. [33] reported similar results; they found that arm lengthening ranging between 1 and 2.5 cm was associated with a better constant score. Shoulder flexion increased until humeral lowering reached a value of 25 mm. Beyond that, shoulder flexion decreased. Other previously published studies also supported this finding. Jobin et al. [34] published a prospective cohort study that included 49 patients who underwent RSA for cuff tear arthropathy. They evaluated deltoid lengthening and medialization of the center of rotation radiographically and correlated with RoM. They demonstrated that deltoid lengthening was significantly correlated with superior shoulder flexion. Lädermann et al. [27] compared 183 patients with arm lengthening and those with arm shortening after RSA; they found that postoperative shoulder flexion was significantly greater after arm lengthening, 145° versus 122°, with a mean difference of 23°. However, a lengthening threshold was not found in their study.

In our study, the more uniform distribution of ACP distalization values allowed us to identify a threshold value of around 35 mm. However, the greater dispersion of DSA values made it impossible to identify a humeral lowering threshold value for this criterion. In a recent study by Berhouet et al. [29], it was revealed that the greater the humeral distalization, the better the abduction, with no threshold effect. However, that study was a computer analysis of passive virtual glenohumeral mobility. These new findings leave us wary of the preoperative planning data provided using the dedicated software and their application during the intervention. In other words, even though lowering the humerus is theoretically beneficial for improving the action of the deltoid and therefore joint mobility, too much humeral distalization is harmful in practice, with less RoM in flexion. It becomes nonsensical to plan more than 25 or 30 mm humeral distalization for RSA. Additionally, it would be difficult to physically reduce the implanted prosthesis at the end of the procedure when the planned excessive humeral lowering is carried out.

The counterproductive threshold effect of excessive humeral distalization, measured using the ACP, was also observed for the other RoM values measured in this study: ABD, ER1, ER2, and IR1. On the other hand, no functional limitation was observed relative to humeral lateralization measured using the ACP. This tended to mainly improve the ER1 but was statistically not significant (*p* = 0.07). In the axial plane, anterior humeral displacement correlates with better RoM in different directions. No threshold effect was observed. These clinical observations are therefore comparable to those reported in a virtual planning study by Berhouet et al. [29]. We propose the following explanation: The humeral displacement induced by RSA in the anteroposterior (6 ± 6 mm) and mediolateral (4.3 ± 3.7 mm) planes is less important than that generated in the frontal plane with humeral distalization. In fact, the soft tissue loading in these different planes is probably less, not exceeding the limits of muscle elongation. We think the rotations are improved by humeral lateralization and anteriorization, as this prevents impingement with the scapular pillar and restores muscular configuration to a more favorable status for the recruitment of the remaining rotator cuff. Thus, virtual preoperative planning may be the most reliable and the least impacted by soft tissues when evaluating shoulder rotation, based on the humeral displacement in the anteroposterior and mediolateral planes [35,36].

The functional consequences of humeral displacement assessed using 2D angle measurements slightly differ from those previously reported with the ACP. Moreover, we found no correlation between the 3D ACP measurements and the 2D angle measurements. The potential deleterious effect on the shoulder flexion of too much distalization, assessed using the DSA, was not observed for other shoulder motions. Lateralization, assessed using the LSA, was favorably related to ER1, ER2, and IR1 but unfavorably to flexion, and had no impact on ABD. The wide dispersion of these different angle values for humeral displacement in our limited study population very likely explains the difficulty in interpreting these results. This may also reflect a limit on these angle measurements being carried out using a 2D image since such measurements are less precise than those in a 3D reference frame. Measuring the 3D humeral displacement via the ACP at the millimeter level allowed for the identification of a threshold value of distalization on the different shoulder motions. This was not the case with the LSA and DSA, which were less accurate. This is further evidence that a 2D parameter is not always suitable for characterizing a 3D movement or position.

The main limitation of this study is the small number of patients included. This contributes to a lack of statistical power and, thus, the inability to identify statistically significant differences. However, this study has several methodological strengths. It was prospective and based on real clinical data. Additionally, it was built around a specific imaging processing protocol for postoperative CT scans, which facilitated the precise measurement of the humeral displacement obtained after RSA implantation and its comparison with that of the preoperative humeral position. In this study, we sought to objectively evaluate the quality of virtual planning information (via the ACP) by comparing it to actual clinical observations. Further investigation with a larger number of patients and long-term follow-up could be carried out to strengthen our findings.

## 5. Conclusions

Beyond the 2D angular or 3D millimeter-level evaluation of humeral displacement after RSA, this study reveals how soft tissue tension impacts the functional results with this type of implant. Determining threshold values for the distalization, lateralization, or even “anteriorization” of the humerus is probably one of the first steps in understanding how to restore the humeral position in order to optimize shoulder joint mobility and prevent deleterious effects. Notably, 3D planning software programs are important elements of this process. The ACP measurement generated with the software used in this study is one of the first parameters designed to explore this issue.

## Figures and Tables

**Table 1:** Preoperative demographic, clinical, and radiological data.

		Age	BMI	Flex	ABD	ER1	ER2	IR1	Constant	CSA	LSA	DSA
**OA group**	Patient 1	81	32.1	30	20	−10	0	Thigh = 0	8	31	92	13
	Patient 2	77	31.6	80	70	5	20	Thigh = 0	25	29	89	8
	Patient 3	74	27.4	80	60	−5	0	Thigh = 0	21	42	87	14
	Patient 4	79	34.4	90	80	0	0	Thigh = 0	23	28	82	−3
	Patient 5	79	29.4	80	80	−10	20	Lumbosacral = 4	12	37	81	3
	Patient 6	68	30.1	90	60	−10	0	Thigh = 0	21	39	100	12
**Mean OA**		76 ± 5 (68–81)	30.8 ± 2.4 (27.4–34.4)	75 ± 23 (30–90)	62 ± 22 (20–80)	−5 ± 6 (−10–5)	7 ± 10 (0–20)	1 ± 2 (0–4)	18 ± 7 (8–25)	34 ± 6 (28–42)	89 ± 7 (81–100)	8 ± 7 (−3–14)
**CTA group**	Patient 7	72	27.1	120	110	10	50	L3 = 6	32	39	125	6
	Patient 8	70	19.5	90	80	0	0	T7 = 10	38	52	41	30
	Patient 9	63	29.4	40	40	30	40	Buttocks = 2	18	39	96	6
	Patient 10	74	28.0	80	70	10	30	Buttocks = 2	24	42	55	5
	Patient 11	71	27.5	110	100	25	50	T7 = 10	34	34	105	18
	Patient 12	73	25.4	150	150	0	20	T7 = 10	47	40	105	18
**Mean CTA**		71 ± 4 (63–73)	26.2 ± 3.5 (19.5–29.4)	98 ± 38 (40–150)	92 ± 38 (40–150)	13 ± 13 (0–30)	32 ± 19 (0–50)	7 ± 4 (2–10)	32 ± 10 (18–47)	41 ± 6 (34–52)	88 ± 33 (41–125)	14 ± 10 (5–30)
***p***		0.04	0.02	0.22	0.12	0.01	0.02	0.006	0.002	0.08	0.96	0.25
**Overall Mean**		73 ± 4 (63–81)	28.5 ± 3 (19.5–34.4)	87 ± 31 (30–150)	77 ± 31 (20–150)	4 ± 10 (−10–30)	19 ± 16 (0–50)	4 ± 4 (0–10)	25 ± 9 (8–47)	38 ± 6 (28–52)	88 ± 24 (41–125)	11 ± 9 (−3–30)

OA: primary osteoarthritis; CTA: cuff tear arthropathy; BMI: body mass index (kg/m^2^); Flex: flexion (°); ABD: abduction (°); ER1: external rotation with elbow at side (°); ER2: external rotation with 90° shoulder abduction; IR1: internal rotation with the highest level reached at the back with the elbow at the side; Constant: Constant–Murley Shoulder Outcome Score; CSA: critical shoulder angle (°); LSA: lateralization shoulder angle (°); DSA: distalization shoulder angle (°).

**Table 2:** Postoperative clinical and radiological data.

		EA	ABD	RE1	RE2	RI1	Constant	CSA	LSA	DSA	ACP Latéralisation	ACP Antériorisation	ACP Distalisation
**OA group**	Patient 1	80	80	0	40	Buttocks = 2	32	28	78	65	8	0	−33
	Patient 2	130	100	10	60	Lumbosacral = 4	67	19	60	34	3	6	−31
	Patient 3	110	100	0	30	Buttocks = 2	66	24	90	46	5	−1	−34
	Patient 4	140	140	20	80	Thigh = 0	67	24	85	56	9	18	−27
	Patient 5	160	160	10	60	T12 = 8	79	23	79	57	8	12	−29
	Patient 6	130	120	5	40	L3 = 6	73	30	84	45	−1	−10	−42
**Mean OA**		125 ± 27 (80–160)	117 ± 29 (80–160)	8 ± 8 (0–20)	52 ± 18 (30–80)	4 ± 3 (0–8)	64 ± 17 (32–79)	25 ± 4(19–30)	79 ± 10 (60–90)	51 ± 11 (34–65)	5 ± 3 (−1–9)	4 ± 8 (−10–18)	−33 ± 4 (−42– −27)
**CTA group**	Patient 7	110	100	10	70	L3 = 6	67	18	87	48	3	8	−38
	Patient 8	100	100	0	40	T12 = 8	60	34	65	44	8	10	−29
	Patient 9	110	90	50	20	T12 = 8	55	47	92	38	10	13	−31
	Patient 10	160	160	10	70	L3 = 6	79	30	73	53	0	11	−30
	Patient 11	130	130	30	70	T7 = 10	66	22	96	38	−1	1	−34
	Patient 12	160	160	0	60	Lumbosacral = 4	72	34	82	58	0	4	−41
**Mean CTA**		128 ± 26 (100–160)	123 ± 31 (90–160)	17 ± 20 (0–50)	55 ± 21 (20–70)	7 ± 2 (4–10)	69 ± 7 (60–79)	31 ± 10 (18–47)	83 ± 12 (65–96)	47 ± 8 (38–58)	3 ± 4 (−1–10)	8 ± 4 (1–13)	−34 ± 4 (−41– −29)
***p***		0.83	0.71	0.31	0.77	0.05	0.57	0.20	0.63	0.49	0.53	0.96	0.21
**Overall mean**		127 ± 27 (80–160)	120 ± 30 (80–160)	12 ± 15 (0–50)	53 ± 20 (20–80)	5 ± 3 (0–10)	66 ± 13 (32–79)	28 ± 8 (18–47)	81 ± 11 (60–96)	49 ± 10 (34–65)	4 ± 4 (−1– 10)	6 ± 6 (−10–18)	−33 ± 4 (−42– −27)

OA: primary osteoarthritis; CTA: cuff tear arthropathy; BMI: body mass index (kg/m^2^); Flex: flexion (°); ABD: abduction (°); ER1: external rotation with elbow at side (°); ER2: external rotation with 90° shoulder abduction; IR1: internal rotation with the highest level reached at the back with the elbow at the side; Constant: Constant–Murley Shoulder Outcome Score; CSA: critical shoulder angle (°); LSA: lateralization shoulder angle (°); DSA: distalization shoulder angle (°); ACP: arm change position (mm).

**Table 3:** Gains in the clinical range of motion (Δ = postoperative measurement − preoperative measurement) and postoperative ACP.

		Δflex	ΔABD	ΔER1	ΔER2	ΔIR1	ΔConstant	ACP lat	ACP ant	ACP dist
**OA group**	Patient 1	50	60	10	40	2	24	8	0	−33
	Patient 2	50	30	5	40	4	42	3	6	−31
	Patient 3	30	40	5	30	2	45	5	−1	−34
	Patient 4	50	60	20	80	0	44	9	18	−27
	Patient 5	80	80	20	40	4	67	8	12	−29
	Patient 6	40	60	15	40	6	52	−1	−10	−42
**Mean OA**		50 ± 16 (30–80)	55 ± 18 (30–80)	13 ± 7 (5–20)	45 ± 18 (30–80)	3 ± 2 (0–6)	64 ± 17 (32–79)	5 ± 3 (−1–9)	4 ± 8 (−10–18)	−33 ± 4 (−42– −27)
**CTA group**	Patient 7	−10	−10	0	20	0	35	3	8	−38
	Patient 8	10	20	0	40	−2	22	8	10	−29
	Patient 9	70	50	20	−20	6		10	13	−31
	Patient 10	80	90	0	40	4	55	0	11	−30
	Patient 11	20	30	5	20	10	32	−1	1	−34
	Patient 12	10	10	0	40	−6	25	0	4	−41
**Mean CTA**		30 ± 36 (−10–80)	32 ± 35 (−10–90)	4 ± 8 (0–20)	23 ± 23 (−20–40)	2 ± 6 (−6–10)	69 ± 7 (60–79)	3 ± 4 (−1–10)	8 ± 4 (1–13)	−34 ± 4 (−41– −29)
***p***		0.25	0.17	0.08	0.10	0.70	0.17	0.53	0.96	0.21
**Overall Mean**		40 ± 29 (−10–80)	43 ± 29 (−10–90)	8 ± 8 (0–20)	34 ± 23 (−20–80)	3 ± 4 (−6–10)	40 ± 14 (22–55)	4 ± 4 (−1– 10)	6 ± 6 (−10–18)	−33 ± 4 (−42– −27)

OA: primary osteoarthritis; CTA: cuff tear arthropathy; Flex: flexion (°); ABD: abduction (°); ER1: external rotation elbow at side (°); ER2: external rotation with 90° shoulder abduction; IR1: internal rotation with the highest level reached at the back with the elbow at the side; Constant: Constant–Murley Shoulder Outcome Score; ACP: Arm Change Position (mm).

## Data Availability

The data used to support the findings of this study are included within the article. Raw data are available from the corresponding author.
